# Impact of Insurance on Readmission Rates, Healthcare Expenditures, and Length of Hospital Stay among Patients with Chronic Ambulatory Care Sensitive Conditions in China

**DOI:** 10.3390/healthcare12171798

**Published:** 2024-09-09

**Authors:** Esthefany Xu Zheng, Xiaodi Zhu, Yi Zhu, Zhenhua Qin, Jiachi Zhang, Yixiang Huang

**Affiliations:** School of Public Health, Sun Yat-sen University, 74, Zhongshan 2nd Road, Guangzhou 510030, China; xuzheng8@mail2.sysu.edu.cn (E.X.Z.); zhuxd9@mail2.sysu.edu.cn (X.Z.); zhuy276@mail2.sysu.edu.cn (Y.Z.); qinzhh5@mail2.sysu.edu.cn (Z.Q.); zhangjch59@mail2.sysu.edu.cn (J.Z.)

**Keywords:** medical insurance, ACSCs, readmission rates, length of hospital stay, health expenses

## Abstract

**Background:** The disparities in healthcare access due to varying insurance coverage significantly impact hospital outcomes, yet what is unclear is the role of insurance in providing care once the patient is in the hospital for a preventable admission, particularly in a weak gatekeeping environment. This study aimed to investigate the association between insurance types and readmission rates, healthcare expenditures, and length of hospital stay among patients with chronic ambulatory care sensitive conditions (ACSCs) in China. **Methods:** This retrospective observational study utilized hospitalization data collected from the Nanhai District, Foshan City, between 2016 and 2020. Generalized linear models (GLMs) were employed to analyze the relationship between medical insurance types and readmission rates, lengths of hospital stay, total medical expenses, out-of-pocket expenses, and insurance-covered expenses. **Results:** A total of 185,384 records were included. Among these, the participants covered by urban employee basic medical insurance (UEBMI) with 44,415 records and urban and rural resident basic medical insurance (URRBMI) with 80,752 records generally experienced more favorable outcomes compared to self-pay patients. Specifically, they had lower readmission rates (OR = 0.57, 95% CI: 0.36 to 0.90; OR = 0.59, 95% CI: 0.42 to 0.84) and reduced out-of-pocket expenses (β = −0.54, 95% CI: −0.94 to −0.14; β = −0.41, 95% CI: −0.78 to −0.05). However, they also experienced slightly longer lengths of hospital stay (IRR = 1.08, 95% CI: 1.03 to 1.14; IRR = 1.11, 95% CI: 1.04 to 1.18) and higher total medical expenses (β = 0.26, 95% CI: 0.09 to 0.44; β = 0.25, 95% CI: 0.10 to 0.40). **Conclusions:** This study found that different types of health insurance were associated with varying clinical outcomes among patients with chronic ambulatory care sensitive conditions (ACSCs) in China. Since the hospitalization of these patients was initially avoidable, disparities in readmission rates, lengths of hospital stay, and medical expenses among avoidable inpatient cases exacerbated the health gap between different insurance types. Addressing the disparities among different types of insurance can help reduce unplanned hospitalizations and promote health equity.

## 1. Introduction

Unplanned hospitalizations remain a pervasive global challenge, demanding urgent attention and solutions. Certain diseases (i.e., hypertension, diabetes, and pneumonia) can be prevented through proactive health management and promotion, thereby reducing the need for hospitalization and treatment. Such diseases are commonly termed ambulatory care sensitive conditions (ACSCs) and are utilized as a quality indicator for primary care in many countries [1,2,3,4]. According to Billings’ definition [1], ACSCs refer to types of conditions that can be prevented through appropriate primary care. This does not imply that individuals could currently avoid hospitalization; rather, it suggests that with less severe conditions, timely primary care could have prevented the escalation to a level that necessitates hospitalization. Well-established gatekeeping systems refer to healthcare systems in which the primary care providers act as gatekeepers, controlling patient access to specialist services and hospital care. For example, in the UK, access to secondary and tertiary care requires a general practitioners’ referral, precluding direct patient access [5]. Countries with robust and structured gatekeeping systems like the Netherlands (0.7%) [6] and Italy (8%) [7] exhibit lower ACSC hospitalization rates. In contrast, ACSC admissions remain significantly higher in countries with weak gatekeeping systems, such as China (10% to 18%) [8,9,10], in which residents have unrestricted access to any healthcare facilities. Residents in China tend to bypass primary care facilities and go to hospitals without a referral [9], due to the uneven distribution of primary care resources between rural and urban areas and the poor performance of community health centers [8]. Hospitals may not be motivated to transfer patients with ambulatory care sensitive conditions and valuable revenue streams to primary care facilities without specific policies or incentives [11,12,13,14]. Therefore, in countries with high rates of unplanned hospitalization, such as China, identifying the factors and adverse outcomes associated with unplanned hospitalization is important for the development of health policies and the allocation of adequate healthcare resources.

China’s healthcare delivery system is organized into two tiers: primary healthcare system and hospital service system [15]. The former is mainly composed of community health service centers, while the latter is mainly composed of three levels of hospitals. Primary care facilities provide public health services, manage residents’ health, and treat common diseases, while first, secondary, and tertiary hospitals offer more specialized and complex care [15,16]. In the absence of a strict gatekeeper system, Chinese residents often bypass community health service centers and go directly to hospitals for treatment. The health insurance system consists mainly of basic medical insurance for urban employees and residents, critical illness insurance, medical assistance programs, and private health insurance. These schemes cover both outpatient and inpatient expenses, with additional reimbursement for costly treatments and financial support for low-income populations [15]. Despite the expansion of insurance coverage, out-of-pocket expenditures remain a large burden for many citizens, particularly in rural areas and low-income groups [17]. Due to China’s household registration system, there exist disparities in healthcare resources across urban and rural areas. The performance of primary care services remains a persistent challenge, while issues related to healthcare quality, efficiency, and equity continue to persist [18].

Previous studies have shown that ACSC admission rates vary by the type of health insurance [19,20]; however, the impact of insurance on hospital outcomes, like readmission rates, remains unclear in cases of preventable admissions in unrestricted access settings. It is foreseeable that the disparate hospitalization outcomes resulting from varying types of insurance will, to some extent, exacerbate the health disparities driven by differences in medical coverage [21,22]. Research across various countries indicates that the type and extent of health insurance coverage significantly affect hospitalization outcomes. Indeed, lack of insurance or self-pay is associated with in-hospital mortality and readmissions [23,24]. This is because these individuals may underutilize healthcare services, leading to adverse hospital outcomes. For high-income countries with universal coverage, such as Germany, disparities in healthcare access and quality persist, resulting in an uneven health status [25]. In Vietnam, patients with health insurance tend to utilize inpatient services more frequently and experience extended hospital stays, indicating that insurance improves access to care [21]. These findings underscore the pivotal role of health insurance in enhancing hospital care and outcomes, reinforcing the necessity for robust insurance systems to improve patient care and address disparities.

Patients who could avoid hospitalization should not be admitted, which can widen the health equity gap due to insurance-based disparities [26,27]. In China, there are certain differences in terms of coverage population, payment standards, reimbursement standards, and service packages between UEBMI, URRBMI, free medical care, medical assistance programs, and private medical insurance [28,29]. UEBMI, targeting urban employees and retirees, covers inpatient and day surgery expenses. Its tiered deductibles are CNY 1000, 500, and 250 for tertiary, secondary, and primary institutions respectively, with reimbursement rates of 95%, 91%, and 87%. The maximum payment limits correlate with enrollment duration and average annual wages [30]. URRBMI, for urban and rural residents, offers similar coverage plus maternity benefits. Its deductibles are CNY 1200, 600, and 300, with rates of 95%, 90%, and 85%. URRBMI’s maximum payment limit is 80% of UEBMI’s [30]. Publicly funded medical care is a state-run social security system that offers free medical and preventive services to state employees through regulated health departments. China is gradually phasing out its free healthcare system, resulting in limited access for most people. Individuals under this scheme enjoy higher reimbursement rates compared to those under UEBMI and URRBMI. Critical illness insurance and medical assistance programs apply to low-income recipients, extremely impoverished individuals, and members of families on the brink of receiving subsistence allowances. Commercial insurance is purchased privately, generally with the highest reimbursement rates and payment standards.

Given the potential burden caused by the insurance-based discrepancies among patients with ambulatory care sensitive conditions in China, we aimed to investigate the impact of health insurance on readmission rates and lengths of hospital stay among hospitalized patients with chronic ambulatory care sensitive conditions. Moreover, in a situation known as a moral hazard, the existence of health insurance makes it easier for individuals to gain benefits (such as more frequent doctor visits and treatments), knowing that the risks and costs are largely borne by the insurance system, explaining the out-of-control health costs. Therefore, we also examined the association between insurance type and healthcare expenditures.

## 2. Methods

### 2.1. Data and Study Population

Our study focused on the Nanhai District, an advanced region within Foshan City, boasting 14 medical centers and a population exceeding 1.74 million as of 2023. This research examined patient hospitalization data spanning from January 2016 through to December 2020, encompassing over 1.62 million hospitalization records. Every record in this repository signifies a unique medical stay or clinical monitoring instance. The comprehensive records, documenting all medical center entries in Nanhai, were obtained from the local Health Commission. This compilation includes facility codes, treatment costs, patient demographics (such as age, gender, and profession), and medical details (procedures coded using ICD-9-CM3 and diagnoses classified as per ICD-10 guidelines).

ACSCs are generally classified into chronic conditions in which effective management prevents exacerbations (such as asthma), acute conditions in which early intervention prevents serious progression (such as pyelonephritis), and vaccine-preventable conditions in which vaccination reduces disease (such as influenza). Given the lack of a global standard for ACSCs’ inclusion and the high burden of chronic diseases in China, we used the list of chronic ACSCs established by the Agency for Healthcare Research and Quality (AHRQ) in the United States. These conditions include diabetes with short-term complications, diabetes with long-term complications, uncontrolled diabetes without complications, diabetes with lower-extremity amputation, chronic obstructive pulmonary disease, asthma, hypertension, heart failure, bacterial pneumonia, and urinary tract infection. Details of the inclusion and exclusion criteria and the ICD codes used to construct the PQIs can be found in the AHRQ guidelines [31].

**Figure 1:**
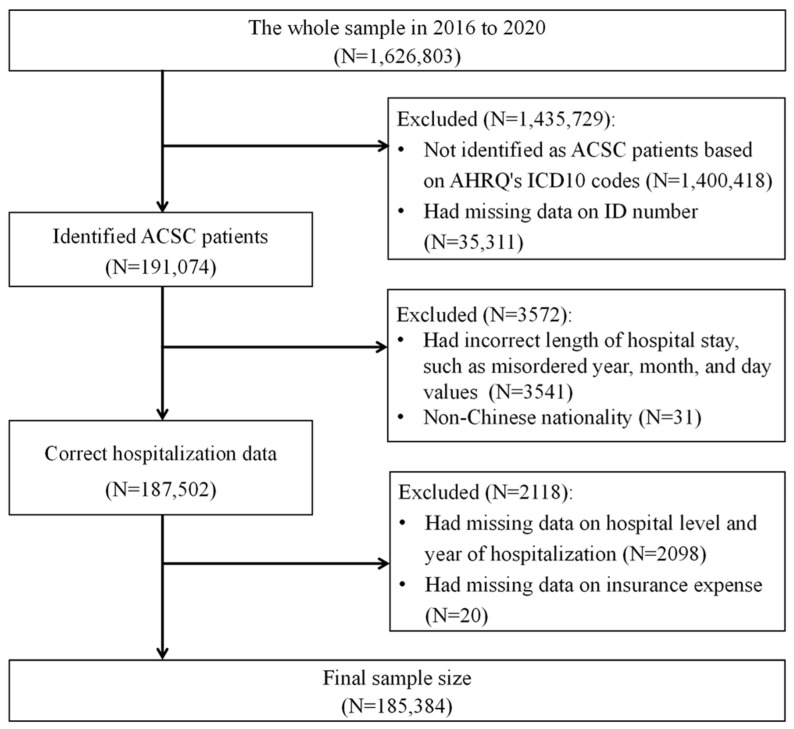
Sample selection steps.

Figure 1 illustrates the derivation of the analytical sample. This study, analyzing patients with ACSCs from 2016 to 2020, involved a detailed sample selection process. Several exclusion criteria were applied to ensure data accuracy and completeness. Initially, the total sample comprised 1,626,803 records. Exclusions were made for records not identified as patients with ACSCs based on AHRQ’s ICD-10 codes and those with missing ID numbers, resulting in 191,074 identified patients with ACSCs. Further exclusions included records with incorrect hospitalization lengths, such as those with misordered year, month, and day values, and records with non-Chinese nationality, reducing the sample to 187,502. The final exclusions involved records missing data on hospital grade and year of medical record and those missing medical expenses, yielding a final sample size of 185,384.

### 2.2. Variables of Interest

The primary outcome variables included readmission rates and lengths of hospital stay, and the second outcome variables included medical costs (total medical expenses, out-of-pocket expenses, and insurance-covered expenses). Readmission rates were defined as the occurrence of any subsequent hospital admission within 30 days after discharge, includes instances in which the patients were rehospitalized for any reason within 30 days following discharge. The lengths of hospitalization were measured in days. The medical costs were divided into total medical expenses, out-of-pocket expenses borne by the patient, and insurance-covered expenses. These outcome variables were further examined in seven specific ACSC categories to determine how different insurance types influenced the readmission rates, lengths of hospital stay, and medical expenses for various chronic conditions.

The patients’ type of health insurance coverage was the primary exposure variable in this study. We categorized insurance types into five groups: urban employee basic medical insurance (UEBMI), urban and rural resident basic medical insurance (URRBMI), free medical service, other types of insurance, and self-pay patients.

To control for potential confounders, several covariates were included in the analysis. The covariates included demographic and clinical factors, encompassing gender, age, marital status, occupation, hospital admission mode (emergency or planned), number of hospital admissions, hospital level, number of hospital beds, surgical procedure, Charlson comorbidity index (CCI), and year of hospitalization.

### 2.3. Statistical Analyses

The characteristics of the patients were summarized using descriptive statistics. For numerical measures, we reported either the average with standard deviation or the median with interquartile range. Categorical variables were expressed as counts and percentages. The association between insurance type and readmission was analyzed using generalized linear models (GLMs) with a binomial distribution. Odds ratios (ORs) and 95% confidence intervals (CIs) were calculated by the GLM. The role of insurance related to length of hospital stay was assessed using a GLM with a Poisson distribution, providing incidence rate ratios (IRRs) and 95% CIs. We also performed GLMs with a gamma distribution to examine the relationship between medical insurance types and healthcare expenses, estimating coefficients and 95% CIs. All expenditure variables in this study were inflation-adjusted to the 2020 Chinese yuan using the gross domestic product (GDP). The robustness of the results was further assessed by separately including individuals aged 18 to 85 years, excluding those covered by the free medical service scheme, or those who died during hospitalization. Analyses were performed with Stata 14.0, and a two-sided *p* value level of 0.05 was considered the threshold for statistical significance.

## 3. Results

### 3.1. Sample Characteristics

A total number of 185,384 hospitalizations records were used for the analyses. The characteristics of the cases, stratified by insurance type, showed significant variations (Table 1). The mean age varied significantly across different insurance groups, with free medical service patients being the youngest (mean age 34.4 years) and those with UEBMI or URRBMI being older (mean ages 62.7 and 53.9 years, respectively). The proportion of male patients was higher in the UEBMI and URRBMI groups (55.4% and 50.9%, respectively). A substantial majority of UEBMI patients were married (85.7%), whereas a significant portion of URRBMI patients were unmarried (26.0%). The highest unemployment rate was observed among URRBMI patients (25.9%), whereas UEBMI and other insurance groups had higher employment rates (24.4% and 35.6%, respectively). Self-pay patients had the highest frequency of multiple hospitalizations (70.2% had more than one hospitalization). Most hospitalizations occurred in secondary hospitals across all groups, with the URRBMI and self-pay patient groups having similar distributions (70.1% and 66.4%, respectively).

### 3.2. Impact of Medical Insurance Type on Readmission Rates, Length of Hospital Stay, and Medical Expenses for Patients with ACSCs

**Figure 2:**
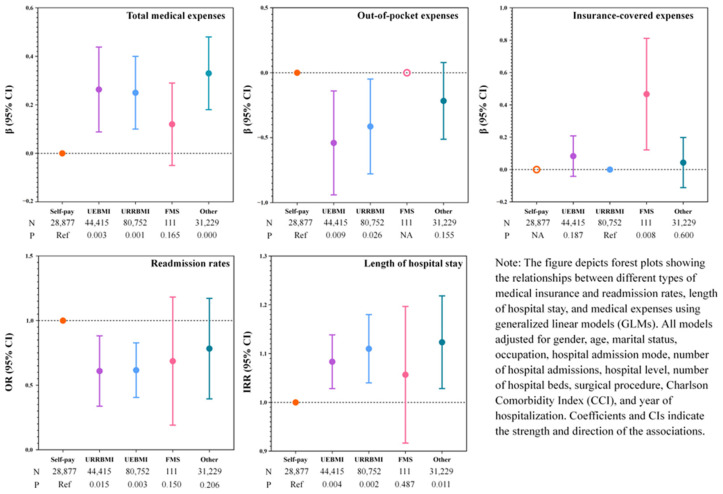
Impact of different types of medical insurance on readmission rates, lengths of hospital stay, and medical expenses of all patients.

Compared with the self-pay patients, patients participating in UEBMI (OR = 0.57, 95% CI: 0.36 to 0.90) and URRBMI (OR = 0.59, 95% CI: 0.42 to 0.84) had lower rates of readmission. In terms of length of stay, there was a significant relationship between the increased length of stay for those covered by UEBMI (IRR = 1.08, 95% CI: 1.03 to 1.14) and URRBMI (IRR = 1.11, 95% CI: 1.04 to 1.18) in comparison with the self-pay patients. In terms of medical expenses, compared to self-pay patients, those in UEBMI had significantly higher total medical expenses (β = 0.26, 95% CI: 0.09 to 0.44) and lower out-of-pocket expenses (β = −0.54, 95% CI: −0.94 to −0.14). Similarly, URRBMI patients had higher total medical expenses (β = 0.25, 95% CI: 0.10 to 0.40) and lower out-of-pocket expenses (β = −0.41, 95% CI: −0.78 to −0.05) (Figure 2; Table S1).

### 3.3. Stratified Analysis of Health Insurance Type and Readmission Rates, Lengths of Hospital Stay, and Medical Expenses for Patients with Specific ACSCs

Further stratified analyses were conducted to explore the relationship between health insurance type and readmission rates, lengths of hospital stay, and medical expenses for specific conditions. Compared to self-pay patients, the readmission rates, lengths of hospital stay, and medical expenses of patients under UEBMI or URRBMI tended to be consistent across different diseases (Table 2). Those covered by UEBMI or URRBM had lower readmission rates, longer lengths of stay, and higher medical expenditures than the self-pay patients.

### 3.4. Sensitivity Analyses

The results did not significantly change after including individuals aged 18 to 85 years or after excluding those covered by the free medical service scheme or those who died during hospitalization (Table S2).

## 4. Discussion

This study examined the impact of health insurance on hospitalization outcomes among patients with ambulatory care sensitive conditions (ACSCs) in China, revealing that different types of healthcare insurance were associated with different readmission rates, lengths of hospital stay, and medical expenses. To some extent, our findings highlight the potential for health disparities and induced demand, depending on insurance type. In China’s current healthcare system, there is an urgent need to reduce insurance disparities to curb unplanned hospital admissions and promote health equity.

The mechanisms underlying these observations were multifaceted. First, health insurance lowers the direct cost of hospitalization for patients, making medical care more accessible [32,33]. Second, health insurance may incentivize providers to recommend more hospitalizations, particularly when the reimbursement rates are favorable [34]. Third, insurance coverage can lead to better preventive care and management of chronic conditions, potentially reducing the need for hospitalization in the long run [35]. This study showed that patients covered by UEBMI and URRBMI had significantly lower readmission rates compared to the self-pay patients. This finding was consistent with previous research emphasizing that comprehensive insurance coverage enhances access to preventive and primary care, thereby reducing the likelihood of hospital readmissions [36]. These results underscore the critical role of robust insurance schemes in mitigating readmission risks by ensuring timely and adequate medical care. Limited access to care, fewer designated hospitals, and disparities in the quality of care are significant factors contributing to this trend [27,37]. These findings emphasize the need for targeted policy interventions to reduce admissions for ambulatory care sensitive conditions. Given that the hospitalization of these patients was initially preventable, disparities in the outcomes for avoidable hospitalizations due to insurance coverage further exacerbated the health disparity among different insurance groups [38,39]. Regarding the length of hospital stay, our findings showed that patients with UEBMI and URRBMI tended to experience slightly longer hospital stays. This finding indicates that insured individuals were more likely to overutilize and waste medical resources. This may be attributed to health professionals providing medical services based on the patients’ insurance status. Additionally, more comprehensive inpatient care and better access to medical resources may contribute to more thorough treatment and recovery processes [40,41].

We also found that out-of-pocket expenditure was significantly lower among those with health insurance, but their total medical expenditure was relatively higher. Due to the differences in enrolment and funding between the urban employee basic medical insurance (UEBMI) and the urban–rural resident basic medical insurance (URRBMI) schemes, insurance coverage can act as a proxy for other socioeconomic factors, such as income, employment status, or overall economic well-being. In many studies [42,43], the insurance coverage is closely associated with the income because higher-income individuals are more likely to have stable employment that provides employer-sponsored insurance, like UEBMI in China. Conversely, lower-income individuals, particularly those who are unemployed or employed in informal sectors, may rely on basic insurance schemes like URRBMI, which offer less comprehensive coverage. A moral hazard arises when individuals with insurance coverage tend to use more healthcare services than they would if they were responsible for the full cost [44]. This occurs because the financial burden of care is partially or fully covered by insurance, leading to the potential overutilization of medical resources. Such overutilization can contribute to the escalation of healthcare costs, especially in systems in which insurance coverage is broad and comprehensive [45,46]. However, from a societal perspective, the induced demand for medical staff can place an economic burden on the entire society, resulting in wasted social security funds and seriously affecting the entire national health system [47,48]. This finding highlights the importance of reducing unplanned hospital admissions, especially for people with health insurance, because of the greater likelihood of inducing healthcare needs [49].

Our stratified analyses indicate that the health insurance coverage significantly influences the readmission rates, lengths of hospital stay, and medical expenses across various diseases. The UEBMI and the URRBMI groups showed consistent effects compared to the self-pay patients. Patients with asthma under UEBMI had high total medical expenses and low out-of-pocket expenses, and patients under URRBMI had high total medical expenses. These findings were consistent with the broader literature on the economic burden of asthma. Health insurance plays a critical role in reducing the financial burden of asthma by reducing out-of-pocket expenses and thereby increasing access to necessary medical services and treatments [50,51]. This is critical for the management of chronic conditions such as asthma, in which consistent and comprehensive care is needed to prevent exacerbations and effectively manage symptoms [52,53]. Patients with CAP insured under UEBMI had lower readmission rates, higher total medical expenses, and significantly reduced out-of-pocket expenses. Patients under URRBMI conditions also had high total medical expenses. Increased age, underlying comorbidities, and greater severity of illness significantly escalate the medical costs for patients with CAP, thereby imposing a heavy economic burden on both patients and the healthcare system [54]. A study conducted in European countries also showed that the high costs for patients with CAP were primarily observed among ICU patients, elderly patients, and those who were hospitalized [55]. Patients with chronic obstructive pulmonary disease (COPD) covered by UEBMI and URRBMI had lower readmission rates, longer lengths of stay, and higher total medical expenses. In addition, patients with COPD had a significant reduction in out-of-pocket expenses under UEBMI. Heitjan et al. showed that, by predicting readmission risk in patients with COPD, healthcare institutions could implement proactive interventions, effectively reducing readmission rates. This approach was crucial for improving patient outcomes and alleviating the burden on the healthcare system [56]. The main reason for the long hospital stays and high medical costs of patients with COPD is the severity and complications of COPD itself [6].

Patients with diabetes had increased total medical costs and decreased out-of-pocket costs under UEBMI and URRBMI. In addition, patient with diabetes under UEBMI had longer lengths of stay and increased insurance-covered expenses. Previous research has shown that patients with diabetes tended to have longer hospital stays compared to patients without diabetes. This may have been due to the complexities in managing diabetes and its complications during hospitalization [57]. Simultaneously, the higher readmission rates and elevated medical expenditures observed among patients with diabetes suggested suboptimal care coordination and disease management [57,58]. For patients with hypertension, UEBMI and URRBMI showed lower readmission rates, longer lengths of stays, and higher total medical expenses. A study found that patients hospitalized for hypertensive emergencies had high readmission rates, longer lengths of stay, and significantly higher medical expenses. These findings highlighted the substantial healthcare burden associated with hypertensive emergencies [59]. Additionally, a study found that the extent of patients’ insurance coverage is a critical factor influencing changes in medical expenses [60]. Patients with heart failure under UEBMI and URRBMI had lower readmission rates and higher total medical expenses, and there were also lower out-of-pocket expenses for patients with UEBMI. Zhang et al. found that, as patient cost-sharing increased, it led to higher out-of-pocket medical expenditures and adverse impacts on health outcomes. This suggests that appropriate control of patient cost-sharing is crucial for containing medical expenses and improving patient health [61]. The evidence suggests that, even with medical insurance coverage, the financial burden on patients with heart failure remains heavy. Consequently, it is essential to implement guideline-recommended therapies to prevent hospital admissions and alleviate the economic burden [62]. Patients with urinary tract infections (UTIs) covered by UEBMI and URRBMI had higher total medical expenses, and their out-of-pocket expenses were significantly lower. Moreover, those under UEBMI had lower readmission rates and those under URRBM had longer lengths of stay. Studies have shown that hospitalization for UTIs can often lead to prolonged stays due to complications and the need for intensive care, particularly in patients with healthcare-associated infections. A significant factor contributing to longer hospital stays was the presence of underlying conditions and the use of urinary catheters, which increased the risk of infections and complications, thereby prolonging hospital stays and escalating healthcare costs. Enhanced monitoring of patients who are at high-risk and the implementation of effective infection control measures could help reduce the incidence of such infections, thereby alleviating the associated healthcare burden [63,64].

### Strengths and Limitations

The strengths of this study included a large sample size and a comprehensive analysis of the impact of different health insurance types on readmission rates, lengths of hospital stay, and medical expenses for patients with ACSCs in China. The use of robust statistical methods to adjust for potential confounders adds to the reliability of the findings. However, this study has limitations, such as potential biases due to unmeasured confounders, the retrospective design, and limited generalizability beyond the study population. In particular, our analysis was limited by the lack of information on the patients’ health status and on important covariates such as smoking, alcohol consumption, and the treatment process of patients. The inclusion of these variables could have been used to adjust for hospitalization-related outcomes. We, thus, controlled the comorbidity index and surgical procedure in the model to minimize the bias caused by the patients’ health status or treatment process. Additionally, the analysis was constrained by the small sample sizes for patients with different ACSCs, which may affect the robustness of the conclusions drawn for these specific diseases.

## 5. Conclusions

This study found that individuals with UEBMI and URRBMI were associated with lower readmission rates, longer hospital stays, and higher medical costs among patients with ambulatory care sensitive conditions in China. To some extent, our findings highlight the potential for health disparities and induced demand depending on insurance type. In China’s current healthcare system, there is an urgent need to address disparities between different types of insurance, to reduce unplanned hospital admissions, and to promote health equity. Policymakers should focus on strengthening the supervision and management of medical insurance funds. Implementing stricter regulations and monitoring mechanisms can help prevent and reduce moral hazard behaviors among healthcare providers. This may include measures such as regular audits and claim reviews. By regulating provider behavior and promoting the rational use of healthcare resources, policymakers can ensure the sustainability of medical insurance funds and improve the efficiency of healthcare delivery.

## Figures and Tables

**Table 1:** Characteristics of participants.

Characteristic	UEBMI (n = 44,415)	URRBMI (n = 80,752)	FMS (n = 111)	Other (n = 31,229)	Self-Pay (n = 28,877)	Total (n = 185,384)	*p* Value
Age; mean (SD)	62.7 (21.3)	53.9 (31.5)	34.4 (33.1)	51.7 (28.6)	43.4 (28.6)	54.0 (29.0)	0.001 ^a^
Age group in years							<0.001 ^b^
≤14	2548 (5.7%)	19,342 (24.0%)	52 (46.85%)	6073 (19.5%)	7499 (26%)	35,514 (19.2%)	
15 to 24	386 (0.9%)	591 (0.7%)	2 (1.8%)	467 (1.5%)	761 (2.6%)	2207 (1.2%)	
25 to 44	3976 (9.0%)	2946 (3.7%)	12 (10.8%)	3240 (10.4%)	3984 (13.8%)	14,158 (7.6%)	
45 to 64	12,055 (27.1%)	14,267 (17.7%)	17 (15.3%)	8112 (26.0%)	8609 (29.8%)	43,060 (23.2%)	
65 to 74	11,046 (24.9%)	17,484 (21.7%)	9 (8.1%)	5900 (18.9%)	3838 (13.3%)	38,277 (20.7%)	
≥75	14,404 (32.4%)	26,122 (32.4%)	19 (17.1%)	7437 (23.8%)	4186 (14.5%)	52,168 (28.1%)	
Year of hospitalization							<0.001 ^b^
2016	8643 (19.5%)	11,472 (14.2%)	32 (28.8%)	8252 (26.4%)	2778 (9.6%)	31,177 (16.8%)	
2017	9256 (20.8%)	11,775 (14.6%)	6 (5.4%)	7645 (24.5%)	3482 (12.1%)	32,164 (17.4%)	
2018	10,488 (23.6%)	17,398 (21.5%)	10 (9.0%)	5790 (18.5%)	7100 (24.6%)	40,786 (22.0%)	
2019	9941 (22.4%)	22,754 (28.2%)	40 (36.0%)	6434 (20.6%)	11,432 (39.6%)	50,601 (27.3%)	
2020	6087 (13.7%)	17,353 (21.5%)	23 (20.7%)	3108 (10.0%)	4085 (14.2%)	30,656 (16.5%)	
Sex							<0.001 ^b^
Male	24,609 (55.4%)	41,094 (50.9%)	71 (64.0%)	16,700 (53.5%)	15,965 (55.3%)	98,439 (53.1%)	
Female	19,806 (44.6%)	39,658 (49.1%)	40 (36.0%)	14,529 (46.5%)	12,912 (44.7%)	86,945 (46.9%)	
Marital status							<0.001 ^b^
Unmarried	3431 (7.7%)	21,004 (26.0%)	55 (49.6%)	6677 (21.4%)	8592 (29.8%)	39,759 (21.5%)	
Married	38,043 (85.7%)	55,507 (68.7%)	50 (45.1%)	22,259 (71.3%)	18,767 (65%)	134,626 (72.6%)	
Unspecified	1976 (4.5%)	1149 (1.4%)	5 (4.5%)	1926 (6.2%)	1016 (3.5%)	6072 (3.3%)	
Widowed/divorced	965 (2.2%)	3092 (3.8%)	1 (0.9%)	367 (1.2%)	502 (1.7%)	4927 (2.7%)	
Occupation							<0.001 ^b^
Unemployed	3181 (7.2%)	20,948 (25.9%)	61 (55.0%)	4240 (13.6%)	6287 (21.8%)	34,717 (18.7%)	
Employed	10,830 (24.4%)	22,845 (28.3%)	17 (15.3%)	11,114 (35.6%)	6641 (23.0%)	51,447 (27.8%)	
Retired	8878 (20.0%)	7044 (8.7%)	6 (5.4%)	3283 (10.5%)	883 (3.1%)	20,094 (10.8%)	
Other	21,526 (48.5%)	29,915 (37.1%)	27 (24.3%)	12,592 (40.3%)	15,066 (52.2%)	79,126 (42.7%)	
Admission route							<0.001 ^b^
Emergency	10,161 (22.9%)	19,220 (23.8%)	24 (21.6%)	4294 (13.8%)	6518 (22.6%)	40,217 (21.7%)	
Outpatient admission	32,422 (73.0%)	58,502 (72.5%)	51 (46.0%)	20,293 (65%)	21,459 (74.3%)	132,727 (71.6%)	
Other	1832 (4.1%)	3030 (3.8%)	36 (32.4%)	6642 (21.3%)	900 (3.1%)	12,440 (6.7%)	
Hospitalization frequency							<0.001 ^b^
1 time	20,450 (46.0%)	37,728 (46.7%)	72 (64.9%)	17,117 (54.8%)	20,279 (70.2%)	95,646 (51.6%)	
2–3 times	12,510 (28.2%)	23,227 (28.8%)	19 (17.1%)	7816 (25.0%)	5727 (19.8%)	49,299 (26.6%)	
>3 times	11,455 (25.8%)	19,797 (24.5%)	20 (18.0%)	6296 (20.2%)	2871 (9.9%)	40,439 (21.8%)	
Hospital level							<0.001 ^b^
Primary	1665 (3.8%)	3941 (4.9%)	2 (1.8%)	2794 (9.0%)	1461 (5.1%)	9863 (5.3%)	
Secondary	25,709 (57.9%)	56,643 (70.1%)	64 (57.7%)	20,742 (66.4%)	16,879 (58.5%)	120,037 (64.8%)	
Tertiary	17,041 (38.4%)	20,168 (25%)	45 (40.5%)	7693 (24.6%)	10,537 (36.5%)	55,484 (29.9%)	
Bed capacity; median (IQR)	600 (508)	550 (145)	500 (125)	608 (138)	600 (828)	586 (117)	0.001 ^a^
CCI; mean (SD)	4 (2.8)	3.4 (2.9)	1.9 (2.9)	2.8 (2.5)	2.2 (2.6)	3.3 (2.8)	0.001 ^a^
Surgical procedure							<0.001 ^b^
None	34,815 (78.4%)	63,205 (78.3%)	74 (66.7%)	27,679 (88.6%)	23,697 (82.1%)	149,470 (80.6%)	
Yes	9600 (21.6%)	17,547 (21.7%)	37 (33.3%)	3550 (11.4%)	5180 (17.9%)	35,914 (19.4%)	
Readmission rates							<0.001 ^b^
None	41,128 (92.6%)	74,360 (92.1%)	105 (94.6%)	29,137 (93.3%)	27,236 (94.3%)	171,966 (92.8%)	
Yes	3287 (7.4%)	6392 (7.9%)	6 (5.4%)	2092 (6.7%)	1641 (5.7%)	13,418 (7.2%)	
Length of hospital stay; mean (SD)	8.32 (6.23)	8.07 (5.87)	7.47 (4.38)	7.81 (5.56)	6.62 (5.25)	7.86 (5.8)	0.001 ^a^
Total medical expenses; median (IQR)	6427.4 (4282.7)	5357.9 (3990.5)	4982.9 (3239.8)	5590.3 (4251.7)	3614.2 (3689.9)	5438.1 (4270.2)	0.001 ^a^
Out-of-pocket expenses; median (IQR)	2364.5 (2446.5)	2444.6 (3257.6)	0 (0)	3296.8 (3089.0)	3614.2 (3689.9)	2770.9 (3241.7)	0.001 ^a^
Insurance-covered expenses; median (IQR)	4710.2 (3497.0)	3565.4 (3245.9)	4982.9 (3239.8)	4075.2 (3207.3)	0 (0)	4039.0 (3435.1)	0.001 ^a^

Abbreviations: UEBMI, urban employee basic medical insurance; URRBMI, urban and rural resident basic medical insurance; FMS, free medical service; SD, standard deviation; IQR, interquartile range; CCI, Charlson comorbidity index. ^a^ Kruskal–Wallis test; ^b^ Pearson chi-square test.

**Table 2:** Stratified analysis of health insurance types and readmission rates, lengths of hospital stay, and medical expenses by patients with different ACSCs.

Disease	Medical Insurance	Readmission Rates OR (95% CI)	Length of Hospital Stay IRR (95% CI)	β (95% CI)		
				Total Medical Expenses	Out-of-Pocket Expenses	Insurance-Covered Expenses
Asthma (n = 2669)	Self-pay	Ref	Ref	Ref	Ref	NA
	UEBMI	0.36 (0.14, 0.95)	1.01 (0.84, 1.22) *	0.31 (0.16, 0.45)	−0.42 (−0.78, −0.06)	0.09 (−0.08, 0.25) *
	URRBMI	0.78 (0.31, 1.97) *	1.07 (0.81, 1.42) *	0.27 (0.07, 0.47)	−0.33 (−0.71, 0.04) *	Ref
	FMS	/	/	/	NA	/
	Other	1.31 (0.65, 2.63) *	1.24 (0.96, 1.60) *	0.40 (0.19, 0.62)	−0.05 (−0.33, 0.23) *	0.05 (−0.22, 0.32) *
CAP (n = 68,815)	Self-pay	Ref	Ref	Ref	Ref	NA
	UEBMI	0.65 (0.50, 0.85)	1.03 (0.97, 1.08) *	0.13 (−0.04, 0.30) *	−0.60 (−0.99, −0.21)	0.01 (−0.10, 0.12) *
	URRBMI	0.63 (0.47, 0.85)	1.07 (1.02, 1.12)	0.18 (0.08, 0.29)	−0.44 (−0.76, −0.12)	Ref
	FMS	0.62 (0.10, 3.70) *	0.96 (0.86, 1.08) *	−0.02 (−0.22, 0.19) *	NA	0.46 (0.21, 0.72)
	Other	0.63 (0.46, 0.86)	1.09 (1.02, 1.17)	0.25 (0.15, 0.35)	−0.19 (−0.44, 0.07) *	−0.04 (−0.15, 0.08) *
COPD (n = 34,579)	Self-pay	Ref	Ref	Ref	Ref	NA
	UEBMI	0.47 (0.25, 0.88)	1.15 (1.03, 1.29)	0.35 (0.12, 0.59)	−0.54 (−1.06, −0.01)	0.13 (−0.04, 0.29) *
	URRBMI	0.52 (0.31, 0.87)	1.20 (1.03, 1.40)	0.31 (0.05, 0.58)	−0.44 (−0.96, 0.08) *	Ref
	FMS	0.37 (0.11, 1.27) *	0.91 (0.73, 1.14) *	0.09 (−0.23, 0.41) *	NA	−0.18 (−0.09, 0.46) *
	Other	0.68 (0.35, 1.32) *	1.14 (0.99, 1.32) *	0.38 (0.13, 0.62)	−0.41 (−0.87, 0.05) *	0.10 (−0.11, 0.31) *
Diabetes (n = 29,961)	Self-pay	Ref	Ref	Ref	Ref	NA
	UEBMI	0.93 (0.61, 1.41) *	1.07 (1.02, 1.11)	0.32 (0.18, 0.46)	−0.55 (−0.85, −0.25)	0.15 (0.02, 0.27)
	URRBMI	0.83 (0.59, 1.17) *	1.03 (0.96, 1.10) *	0.22 (0.09, 0.35)	−0.52 (−0.8, −0.24)	Ref
	FMS	/	1.03 (0.75, 1.43) *	0.03 (−0.19, 0.24) *	NA	0.14 (−0.15, 0.42) *
	Other	0.85 (0.51, 1.40) *	1.11 (1.01, 1.23)	0.31 (0.13, 0.50)	−0.29 (−0.55, −0.03)	0.04 (−0.11, 0.19) *
Hypertension (n = 29,746)	Self-pay	Ref	Ref	Ref	Ref	NA
	UEBMI	0.39 (0.19, 0.82)	1.25 (1.17, 1.33)	0.54 (0.35, 0.72)	−0.25 (−0.65, 0.15) *	0.23 (0.03, 0.43) *
	URRBMI	0.40 (0.23, 0.69)	1.24 (1.13, 1.36)	0.42 (0.22, 0.62)	−0.23 (−0.65, 0.19) *	Ref
	FMS	/	1.46 (0.86, 2.46) *	0.30 (0.00, 0.61)	NA	0.26 (−0.12, 0.64) *
	Other	0.67 (0.34, 1.32) *	1.29 (1.18, 1.42)	0.53 (0.28, 0.78)	−0.06 (−0.36, 0.25) *	0.17 (−0.09, 0.43) *
Heart failure (n = 10,982)	Self-pay	Ref	Ref	Ref	Ref	NA
	UEBMI	0.70 (0.51, 0.96)	1.15 (1.00, 1.32) *	0.36 (0.05, 0.67)	−0.64 (−1.13, −0.15)	0.03 (−0.09, 0.15) *
	URRBMI	0.70 (0.53, 0.91)	1.12 (0.97, 1.30) *	0.38 (0.06, 0.69)	−0.4 (−0.92, 0.12) *	Ref
	FMS	/	1.05 (0.76, 1.45) *	−0.09 (−0.45, 0.27) *	NA	−0.22 (−0.47, 0.02) *
	Other	0.69 (0.53, 0.89)	1.16 (1.03, 1.30)	0.62 (0.28, 0.95)	0.01 (−0.38, 0.40) *	0.12 (−0.06, 0.30) *
UTIs (n = 8632)	Self-pay	Ref	Ref	Ref	Ref	NA
	UEBMI	0.60 (0.38, 0.96)	1.04 (1.00, 1.09) *	0.27 (0.15, 0.39)	−0.44 (−0.76, −0.11)	0.05 (−0.04, 0.15) *
	URRBMI	0.58 (0.31, 1.10) *	1.10 (1.05, 1.16)	0.25 (0.13, 0.36)	−0.34 (−0.64, −0.03)	Ref
	FMS	/	1.38 (1.01, 1.90)	0.43 (0.16, 0.70)	NA	0.72 (0.40, 1.05)
	Other	0.56 (0.26, 1.21) *	1.03 (0.85, 1.25) *	0.21 (−0.02, 0.44) *	−0.19 (−0.41, 0.03) *	0.03 (−0.15, 0.22) *

* *p* > 0.05. Abbreviations: OR, odds ratio; CI, confidence interval; IRR, incidence rate ratio; β, coefficients; NA, these individuals did not incur this expense; CAP, community-acquired pneumonia; COPD, chronic obstructive pulmonary disease; UTIs, urinary tract infections; UEBMI, urban employee basic medical insurance; URRBMI, urban resident basic medical insurance; FMS, free medical service; Ref, reference. All models adjusted for gender, age, marital status, occupation, hospital admission mode, number of hospital admissions, hospital level, number of hospital beds, surgical procedure, Charlson comorbidity index (CCI), and year of hospitalization.

## Data Availability

Data are contained within the article.

## Supplementary Materials

The following supporting information can be downloaded at https://www.mdpi.com/article/10.3390/healthcare12171798/s1: Table S1. Impact of different types of medical insurance on readmission rates, length of hospital stay and healthcare expenditures. Table S2. Impact of medical insurance on readmission rates, length of hospital stay and healthcare expenditures: sensitivity analysis results.
