# Supplementary material for: Impact of Insurance on Readmission Rates, Healthcare Expenditures, and Length of Hospital Stay among Patients with Chronic Ambulatory Care Sensitive Conditions in China
Source: Healthcare (Basel). 2024 Sep 9;12(17):1798. doi: 10.3390/healthcare12171798 (PMC11395110; doi:10.3390/healthcare12171798)
Supplement: Supplementary file 1 [file healthcare-12-01798-s001.zip › healthcare-3143117-supplementary.pdf]

SUPPLEMENTARY MATERIAL

Impact of Insurance on Readmission Rates, Healthcare Expenditures, and Length of Hospital Stay among Patients with Chronic Ambulatory Care Sensitive Conditions in China

Table S1. Impact of different types of medical insurance on readmission rates, lengths of hospital stay and healthcare expenditures

Medical insurance	Logit Regression Readmission rates OR (95% CI)	Poisson Regression Length of hospital stay IRR (95% CI)	Gamma Regression		
			Total medical expenses β (95% CI)	Out-of-pocket expenses β (95% CI)	Insurance-covered expenses β (95% CI)
Self-pay	Ref	Ref	Ref	Ref	NA
UEBMI	0.57 (0.36, 0.90)	1.08 (1.03, 1.14)	0.26 (0.09, 0.44)	−0.54 (−0.94, −0.14)	0.08 (−0.04, 0.21)*
URRBMI	0.59 (0.42, 0.84)	1.11 (1.04, 1.18)	0.25 (0.10, 0.40)	−0.41 (−0.78, −0.05)	Ref
FMS	0.57 (0.26, 1.23)*	1.05 (0.92, 1.20)*	0.12 (−0.05, 0.29)*	NA	0.47 (0.12, 0.81)
Other	0.72 (0.43, 1.20)*	1.12 (1.03, 1.22)	0.33 (0.18, 0.48)	−0.22 (−0.51, 0.08)*	0.04 (−0.11, 0.20)*

* $p>0.05$

Abbreviations: OR, odds ratio; CI, confidence interval; IIR, incidence rate ratio; β, coefficients; NA, these individuals did not incur this expense; UEBMI, urban employee basic medical insurance; URRBMI, urban resident basic medical insurance; FMS, free medical service; Ref, reference. All models adjusted for gender, age, marital status, occupation, hospital admission mode, number of hospital admissions, hospital level, number of hospital beds, surgical procedure, Charlson comorbidity index (CCI), and year of hospitalization.

Table S2. Impact of medical insurance on readmission rates, lengths of hospital stay and healthcare expenditures: sensitivity analysis results

Items	Medical insurance	Readmission rates OR (95% CI)	Length of hospital stay IRR (95% CI)	β (95% CI)		
				Total medical expenses	Out-of-pocket expenses	Insurance-covered expenses
Age group in 18 to 85 (n=135,402)	Self-pay	Ref	Ref	Ref	Ref	NA
	UEBMI	0.59 (0.35, 0.98)	1.10 (1.05, 1.17)	0.31 (0.14, 0.47)	−0.54 (−0.93, −0.14)	0.10 (−0.03, 0.23)*
	URRBMI	0.61 (0.41, 0.92)	1.12 (1.04, 1.21)	0.26 (0.09, 0.44)	−0.46 (−0.84, −0.07)	Ref
	FMS	0.21 (0.05, 0.84)	0.97 (0.78, 1.20)*	0.03 (−0.16, 0.23)*	NA	0.13 (−0.04, 0.31)*
	Other	0.69 (0.41, 1.17)*	1.13 (1.01, 1.27)	0.36 (0.16, 0.56)	−0.26 (−0.59, 0.06)*	0.08 (−0.09, 0.25)*
Removing the free medical service scheme (n=111)	Self-pay	Ref	Ref	Ref	Ref	NA
	UEBMI	0.57 (0.36, 0.90)	1.08 (1.03, 1.14)	0.26 (0.09, 0.44)	−0.54 (−0.94, −0.14)	0.08 (−0.04, 0.21)*
	URRBMI	0.59 (0.42, 0.84)	1.11 (1.04, 1.18)	0.25 (0.10, 0.40)	−0.41 (−0.78, −0.05)	Ref
	Other	0.72 (0.43, 1.20)*	1.12 (1.03, 1.22)	0.26 (0.09, 0.44)	−0.22 (−0.51, 0.08)*	0.04 (−0.11, 0.20)*
Excluding deceased patients (n=815)	Self-pay	Ref	Ref	Ref	Ref	NA
	UEBMI	0.57 (0.36, 0.90)	1.08 (1.03, 1.14)	0.27 (0.09, 0.44)	−0.53 (−0.93, −0.14)	0.09 (−0.04, 0.21)*
	URRBMI	0.59 (0.42, 0.84)	1.11 (1.04, 1.18)	0.25 (0.10, 0.40)	−0.41 (−0.77, −0.05)	Ref
	FMS	0.62 (0.29, 1.32)*	1.04 (0.91, 1.19)*	0.12 (−0.04, 0.29)*	NA	0.47 (0.11, 0.82)
	Other	0.72 (0.43, 1.20)*	1.12 (1.03, 1.22)	0.33 (0.18, 0.49)	−0.21 (−0.51, 0.08)*	0.04 (−0.11, 0.20)*

* $p>0.05$

Abbreviations: OR, odds ratio; CI, confidence interval; IIR, incidence rate ratio; β, coefficients; NA, these individuals did not incur this expense; UEBMI, urban employee basic medical insurance; URRBMI, urban resident basic medical insurance; FMS, free medical service; Ref, reference. All models adjusted for gender, age, marital status, occupation, hospital admission mode, number of hospital admissions, hospital level, number of hospital beds, surgical procedure, Charlson comorbidity index (CCI), and year of hospitalization.
